# Birthweight After Frozen Embryos Formed on the Fifth Day Versus the Sixth Day: A Retrospective Analysis Including 17,127 Singleton Newborns

**DOI:** 10.3389/fendo.2022.868335

**Published:** 2022-05-24

**Authors:** Junlan Yang, Ze Wang, Hairu Cao, Lu Liu, Qiaona Yuan, Haiyan Xu, Rong Tang

**Affiliations:** ^1^ Center for Reproductive Medicine, Cheeloo College of Medicine, Shandong University, Jinan, China; ^2^ Shandong Provincial Hospital Affiliated to Shandong First Medical University, Shandong First Medical University, Jinan, China

**Keywords:** frozen embryo transfer, blastocyst, birth weight, SGA, LGA

## Abstract

**Background:**

Transferring blastocysts frozen on day 6 (D6) may adversely affect the pregnancy rate compared with day 5 (D5). Moreover, it remains unclear whether delayed embryo transfer affects neonatal birth weight.

**Methods:**

A retrospective cohort study consisting of 17,127 singleton births from single frozen embryo transfer (FET) cycles, between January 2011 and January 2020, was performed including 14,166 blastocysts frozen on D5 and 2,961 on D6. The primary outcomes of this study were neonatal birth weight and incidence of small for gestational age (SGA), large for gestational age (LGA), low birth weight (LBW), and macrosomia.

**Results:**

The mean neonatal birth weight in the D5 group (3.47 ± 0.49 kg) was significantly higher compared with the D6 group (3.45 ± 0.50 kg), although the discrepancy was only 0.02 kg. Multiple linear regression analysis for birth weight between the two groups showed no statistically significant difference (β= -0.01 t= -1.218; P>0.05). Logistic regression analysis revealed that the risks of SGA (OR 1.166; 95%CI, 0.911-1.491; P>0.05), LGA (OR 0.917; 95%CI, 0.831-1.012; P>0.05), LBW (OR 1.192; 95%CI, 0.926-1.533; P>0.05), and macrosomia (OR 0.975; 95%CI, 0.864-1.100; P>0.05) were similar in the two groups after adjusting for confounders.

**Conclusions:**

In the FET cycle, the neonatal birth weight and incidence of LGA, SGA, LBW, or macrosomia were similar between the D5 and D6 groups, suggesting that delayed blastocyst transfer would not affect the neonatal birth weight.

## Introduction

Embryo transfer (ET) at the blastocyst stage has been widely recommended in assisted reproductive technology (ART), especially in the single ET program. Theoretically, prolonged in-vitro culture to blastocyst from the cleavage stage allows for better selection of the implantation potential of the embryo, thereby improving the pregnancy rate. Moreover, single ET reduces the incidence of multiple pregnancy rates, which is associated with a higher risk of maternal and neonatal complications (1). Blastocysts are usually formed on the fifth day (D5) of in-vitro culture, while blastulation of some embryos can be delayed to the sixth day (D6) or even later. The embryonic development rate is suggested as an essential indicator of reproductive outcomes. It has been reported that D6 blastocysts generally have a higher rate of aneuploidy than D5 blastocysts (2). Irani et al. have observed that the transfer of D5 euploid blastocysts results in higher rates of clinical pregnancy and live birth compared with those at D6 in preimplantation genetic testing (PGT) cycles (3). A proposed explanation is that the superior implantation potential of D5 blastocysts can be attributed to metabolic or epigenetic factors that may differ in the embryos at different development stages.

The birth weight of neonates has long been regarded as an indicator of the offspring’s health. Chiavaroli et al. have reported that infants born with small-for-gestational-age (SGA) or large-for-gestational-age (LGA) show adverse cardio-metabolic profiles during childhood and adolescence, leading to an increased risk of cardiovascular diseases later in life (4). In addition, LGA is associated with a high risk of offspring obesity and depression (5, 6). Previous findings have indicated that frozen embryo transfer (FET) is associated with a higher birthweight and an increased risk of delivering LGA babies as compared to fresh embryo transfer (7, 8), implying that the process of cryopreservation can adversely affect the embryo quality and developmental potential. Moreover, FET at the blastocyst stage is associated with higher birthweight and an increased risk of LGA compared with the cleavage stage (9, 10). However, limited studies have compared the perinatal outcomes after the transfer of frozen-thawed blastocysts formed on D5 and D6, and the results remain controversial (11, 12). In the present study, we aimed to explore the effect of frozen-thawed blastocyst, formed at different developmental stages, on perinatal outcomes.

## Materials and Methods

### Study Design and Participants

We performed a retrospective cohort study including 17,127 singleton live births after the transfer of frozen-thawed blastocysts from January 2011 to January 2020. Live birth was defined as a birth exhibiting life signs with ≥24 gestational weeks (13). This study was approved by the Institutional Review Board of the Centre for Reproductive Medicine affiliated with Shandong University. Inclusion criteria were as follows: (1) age ≤ 40 years; (2) body mass index (BMI) ≤35 kg/m2; and (3) the transfer of frozen-thawed blastocysts formed on D5 or D6. The exclusion criteria were as follows: (1) women with uterine malformations or intrauterine adhesions; (2) women diagnosed hypertensive disorders, chronic diabetes, and gestational diabetes mellitus (GDM). Hypertensive disorders included gestational hypertension (blood pressure ≥140/90 mm Hg after 20 weeks of gestation), preeclampsia, and eclampsia; and (3) frozen embryos that had undergone the re-cryopreservation process or preimplantation genetic testing.

### Procedures

*In vitro* fertilization (IVF) or intracytoplasmic sperm injection (ICSI) was performed after oocyte collection by the routine procedure. On D3 after insemination, embryos were observed and graded by morphological criteria based on the number and size of the blastomere.

The extended culture of embryos was determined according to the quantity and quality of cleavage-stage embryos, in combination with the request of patients and the evaluation of clinicians. On D3, embryos were removed from the cleavage medium and placed in the blastocyst medium, followed by incubation to D5 or D6, and even D7 until the blastocyst formed. The blastocyst quality was assessed based on the Gardner score system (14), which was frozen if it developed up to 4BC by vitrification.

Endometrial preparation was carried out under natural cycles, stimulation cycles, and artificial cycles, depending on the clinician’s discretion. In a natural ovulation regimen, detection of spontaneous or triggered ovulation was the indication of timing for FET. Oral dydrogesterone was administered for luteal phase support after ovulation (Duphaston, 20-30 mg, or Utrogestan, 200-300mg, once a day). The single frozen-thawed blastocyst was transferred on the 5th day after ovulation. If pregnancy was achieved, dydrogesterone was continued until the 10th week of gestation. In the artificial cycle, oral estradiol (Progynova, 4-8 mg) was taken once a day from day 1-3 of the menstrual cycle. Oral (dydrogesterone, 20 mg, once a day) and transvaginal progesterone (Crinone 8% vaginal gel, 90 mg, once a day) or (dydrogesterone, 40 mg, once a day) and transvaginal progesterone (Utrogestan, 200mg, once a day) was added when the endometrium reached 7 mm or more. Single FET was performed on the sixth day of the progesterone exposure. If pregnancy occurred, estrogen supplement was stopped at the 8th week of gestation and progesterone support was continued until the 10th week of gestation. For stimulation cycles, letrozole or human menopausal gonadotropin (HMG), either alone or in combination, was given from day 3-5 of the menstrual cycle. Ultrasound monitoring was repeated every 1-3 days according to follicle growth until ovulation triggering. When the dominant follicle reached a diameter of 17–20 mm, human chorionic gonadotropin (hCG) at a dosage of 5,000-10,000 IU was administered to trigger ovulation. Other procedures were the same as the natural cycle.

### Outcome Measures and Definitions

The primary outcomes included birth weight (including absolute birth weight), SGA [defined as weighing less than the 10th percentile of birth weight (15)], LGA (defined as weighing more than the 90th percentile of birth weight), LBW (defined as birth weight less than 2,500 g), and macrosomia (defined as infant birth weight more than 4,000 g). The secondary outcomes were cesarean delivery, gestational age [defined as pregnancy weeks from the 19th day before FET to delivery (16)], and preterm birth (defined as a baby born at less than 37 weeks of gestation).

### Statistical Analysis

Baseline characteristics and neonatal outcomes were compared between the study groups by t-test (for continuous variables) or chi-square test (for categoric variables). A multiple linear regression analysis was performed to assess the relationship between the blastocyst development rate and birth weight with adjustment for potential confounding factors, including maternal age (continuous variable), parity (binary variable), basal follicle-stimulating hormone (FSH, continuous variable), FET regimens for endometrial preparation (binary variable), fertilization method (binary variable), embryo quality (binary variable), and developmental stage (binary variable). Logistic regression analysis was performed to evaluate the effect on the incidence of LGA/SGA after adjustment for the potential confounding factors. Odds ratios (OR) and 95% confidence intervals (CI) were used. P<0.05 was considered statistically significant. All statistical analyses were performed in SPSS version 22 software.

## Results

### Baseline Characteristics

A total of 17,127 women who met the study inclusion criteria were enrolled, including 14,166 cases in the D5 group and 2,961 cases in the D6 group. **Table 1** presents the baseline characteristics. No difference was observed in maternal BMI, gravidity, infertility cause, and endometrial thickness between the two groups. Maternal age, parity, basal FSH level, endometrial preparation protocols, and fertilization method showed significant differences between the two groups (P<0.05). These parameters were then adjusted as potential confounders in the logistic regression (**Table 3**) and multiple linear regression analyses (**Table 4**).

**Table 1:** Demographics and cycle characteristics.

Characteristics	D5 (n=14166)	D6 (n=2961)	P
**Baseline demographics**			
Maternal age, (year)	30.14 (±3.79)	30.82 (±3.98)	<0.05
Maternal BMI, (kg/m^2^)	23.11 (±3.39)	23.11 (±3.46)	0.99
Nulligravida, n (%)	8038 (56.7)	1643 (55.5)	0.21
Nulliparity, n (%)	11756 (83.0)	2298 (77.6)	<0.05
Causes of infertility, n (%)			0.07
Tubal factors	8654 (61.1)	1750 (59.1)	
Anovulatory factors	385 (2.7)	74 (2.5)	
Unexplained factors	390 (2.8)	95 (3.2)	
Male factors	2368 (16.7)	557 (18.8)	
Combined factors^a^	2229 (15.7)	456 (15.4)	
Others^b^	140 (1.0)	29 (1.0)	
Basal FSH level (IU/L)	6.28 (±1.72)	6.81 (±2.26)	<0.05
Fasting blood glucose level (mmol/L)	5.21 (±0.39)	5.23 (±0.36)	0.09
Fresh **cycle characteristics**			
Fertilization method, n (%)			<0.05
IVF	10290 (72.6)	1807 (61.0)	
ICSI	3876 (27.4)	1154 (39.0)	
Embryo quality, n(%)			<0.05
Good	12422 (87.7)	1926 (65.0)	
Poor	1744 (12.3)	1035 (35.0)	
**FET cycle characteristics**			
endometrial preparation protocols, n (%)			<0.05
Natural cycle	7898 (55.8)	1739 (58.7)	
Stimulated cycle	1344 (9.5)	301 (10.2)	
Artificial cycle	4924 (34.8)	921 (31.1)	
Endometrial thickness, (cm)	0.98 (±0.16)	0.98 (±0.16)	0.17

Data are presented as mean ± SD, median (IQR) or n (%).

a Combined factors, both with Male and female factors.

b Others, advanced age and/or diminished ovarian function.

### Neonatal Outcomes

**Table 2** presents the neonatal outcomes based on blastocyst development rate. The mean birth weight was 3.47 ± 0.49 kg and 3.45 ± 0.50 kg in the D5 group and D6 group, respectively, showing a significant difference between the two groups, although the discrepancy was only 0.02 kg. Regarding the other neonatal outcomes, no differences were observed between the two groups in terms of the incidence of SGA (2.6% vs. 3.0%; P>0.05), LGA (23.9% vs. 22.7%; P>0.05), LBW (2.5% vs. 2.9%; P>0.05), macrosomia (14.0% vs. 13.6%; P>0.05), and preterm birth (5.0% vs. 5.4%; P>0.05). Notably, the rate of cesarean section was higher following the transfer of blastocysts vitrified on D6 compared with D5 (68.4% vs. 74.3%; P<0.05). Logistic regression was performed after adjustment for the effects of potential confounding factors on the risks of SGA, LGA, LBW, and macrosomia (**Table 3**). There were also no statistically significant differences in the risks of SGA (OR 1.166; 95%CI, 0.911-1.491; P>0.05), LGA (OR 0.917; 95%CI, 0.831-1.012; P>0.05), LBW (OR 1.192; 95%CI, 0.926-1.533; P>0.05), macrosomia (OR 0.975; 95%CI, 0.864-1.100; P>0.05), and preterm birth (OR 1.104; 95%CI, 0.917-1.329; P>0.05) between the two groups. Regression analysis showed that women who received D6 embryos were more likely to undergo cesarean section (OR 1.262; 95%CI, 1.148-1.387; P<0.05) compared with those who received D5 embryos. Following multiple linear regression, no difference was observed between the D5 and D6 groups in terms of birth weight (β= -0.01 t= -1.218; P>0.05), as shown in **Table 4**.

**Table 2:** Neonatal outcomes transferred from blastocysts frozen at day 5 vs day 6.

Outcomes	D5	D6	P
Cesarean section, n (%)	9683 (68.4)	2199 (74.3)	<0.05
Gestational age (wk)	39.23 (±1.57)	39.19 (±1.58)	0.14
Gestational age, n (%)			0.612
24-28w	10 (0.1)	2 (0.1)	
28-37w	704 (5.0)	156 (5.3)	
37-42w	13415 (94.7)	2792 (94.3)	
≥42w	37 (0.3)	11 (0.4)	
Birth weight (kg)	3.47 (±0.49)	3.45 (±0.50)	<0.05
Z-scores	0.62 (±1.07)	0.58 (±1.07)	0.067
Male gender, n (%)	7635 (53.9)	1600 (54.0)	0.89
Preterm birth (<37 weeks), n (%)	708 (5.0)	160 (5.4)	0.36
Low birthweight, n (%)	352 (2.5)	85 (2.9)	0.23
SGA^c^, n (%)	363 (2.6)	90 (3.0)	0.14
LGA^d^, n (%)	3392 (23.9)	673 (22.7)	0.16
Macrosomia, n (%)	1985 (14.0)	403 (13.6)	0.57

Data are expressed as mean ± SD, or number (percent).

c SGA, small for gestational age.

d LGA, large for gestational age.

**Table 3:** Logistic regression analysis of potential confouders^e^ for neonatal outcomes from frozen blastocyst at day 5 or day 6.

Outcomes	OR (95% CI)	P	Adjust OR (95% CI)	P
Cesarean section	1.336 (1.221-1.461)	<0.05	1.262 (1.148-1.387)	<0.05
Preterm birth (<37 weeks)	1.086 (0.910-1.295)	0.360	1.104 (0.917-1.329)	0.297
Low birthweight	1.160 (0.912-1.475)	0.226	1.192 (0.926-1.533)	0.173
SGA	1.192 (0.943-1.507)	0.141	1.166 (0.911-1.491)	0.222
LGA	0.943 (0.850-1.027)	0.157	0.917 (0.831-1.012)	0.083
Macrosomia	0.967 (0.862-1.085)	0.566	0.975 (0.864-1.100)	0.678

e potential confouders including maternal age (continuous variable)，parity (binary variable), basal follicle-stimulating hormone (continuous variable), FET regimens for endometrial preparation (binary variable), fertilization method (binary variable), embryo quality(binary variable), developmental stage (binary variable).

**Table 4:** Multiple regression analysis of potential confounders associating with neonatal birth weight.

	Unstandardized coefficients		Standardized coefficients		
	B	Std. error	β	t	P
Model					
(constant)	3.547	0.037		97.167	<0.05
Maternal age, (year)	-0.001	0.001	-0.009	-1.016	0.309
Nulliparity	0.041	0.01	0.035	4.143	<0.05
Basal FSH level (IU/L)	-0.006	0.002	-0.026	-3.349	<0.05
Fertilization method	-0.007	0.008	-0.007	-0.864	0.388
Embryo quality	-0.017	0.01	-0.014	-1.749	0.08
Endometrial preparation protocols	0.02	0.004	0.041	5.304	<0.05
Blastocyst (Day5/6)	-0.012	-0.009	-0.01	-1.218	0.223

## Discussion

To the best of our knowledge, our current study was, to date, the largest investigation on the effect of embryonic development duration before freezing at the blastocyst stage on neonatal birth weight after FET. The results of this retrospective cohort study indicate that embryonic development duration before freezing at the blastocyst stage did not affect neonatal birth weight, confirming the findings of previous small-scale retrospective studies (11, 17). Meanwhile, in our present study, no association was found between delayed blastocysts and increased risks of SGA, LGA, LBW, and macrosomia, suggesting that delayed blastulation did not adversely affect the birth weight of offspring in FET cycles. Hiraoka et al. have reported that there are no significant differences in gestational age, preterm delivery rate, and birth weight when the embryonic development duration is different. However, only 71 deliveries are included in their study (17). Wang et al. have also reported that the gestational age and birth weight show no significant difference between the D5 and D6 groups (11), with 515 cases in their study. Furthermore, both the above-mentioned studies are undermined by the presence of twins and the limited population size and none of them have reported the outcomes with adjustment for gestational age and gender. Our results show that there was no difference in the risks of LGA and SGA in singletons born from the blastocysts frozen on D6 compared with D5 after adjustment for gender and gestational age, which was consistent with previous findings (11). Regarding birth weight, there was no significant difference after multiple linear regression.

It remains unclear whether the blastocyst development rate affects the perinatal outcomes. Several studies have found that slow-growing blastocysts with delayed expansion on D6 have no impact on clinical results after transferring good-quality embryos, suggesting that delayed blastulation is not related to viability (18, 19). In Yang’s study, the blastocyst quality is a crucial factor that affects pregnancy outcomes (18). Meanwhile, some other studies have indicated that implantation rate, clinical pregnancy rate, and live birth rate derived from the D5 group are higher compared with the D6 group (20–22). Ferreux et al. have speculated that the difference in clinical outcomes is ascribed to chromosomal abnormalities (20). Previous studies have reported a higher aneuploidy rate in D6 blastocysts (2, 23). These findings indicate that there are significant differences in reproductive potential between women undergoing D5 and D6 blastocyst transfers. The differences are partly ascribed to a higher aneuploidy rate among D6 blastocysts. Some studies have reported that D5 blastocysts exhibit significantly higher mitochondrial DNA (mtDNA) levels compared with the D6 group. Moreover, aneuploid blastocysts have higher amounts of mtDNA than euploid blastocysts (24, 25). Animal studies have reported a higher incidence rate of apoptosis in delayed blastocysts compared with blastocysts transferred early. Moreover, the gene expression profile and diameter of blastocysts depend on the developmental stage of the blastocysts (26). In addition, Hashimoto et al. have shown that the incidence of spindle abnormalities is higher in growth-retarded embryos. However, no significant differences are found in the birth weight and gestational age between the groups receiving embryos vitrified on D5 and D6. They conclude that most blastomeres with abnormal spindles are eliminated before implantation (27).

Wang et al. have reported a higher proportion of LBW in the D5 group compared with the D6 group. They consider that the increased birth weight is associated with the extended culture (11). Inconsistent with Cai’s study, they have reported a significantly higher risk of LGA in singletons born after delayed blastocyst transfer on D6 (12). Ferreux’s study has also found that the birth weight of neonates derived from the D5 group is less compared with the D6 group. They have proposed that extended *in vitro* culture contributes to the heavier birth weight. Nevertheless, they have not reported the outcomes with adjustment for gestational age and gender (20). Existing data on the effect of culture duration on neonatal birth weight are conflicting. Some studies have reported that compared with cleavage embryos, blastocyst transfer tends to have a higher mean birth weight and an increased proportion of LGA (9, 10, 28). An animal study has demonstrated that the effects of delayed blastulation and extended culture on blastocysts can be cumulative (26). However, others have shown that culture duration is not correlated with neonatal birth weight (29, 30). Du et al. have reported retarded embryos do not result in a high risk of LBW, congenital malformations, and early neonatal death (31). Our data further indicated that there was no difference in neonatal weight with the increased duration to 6 days compared with 5 days for blastulation.

Previous studies have shown that the neonatal birth weight may be affected by circumstances of embryonic development, such as endometrial receptivity (32, 33), different culture medium (16, 34), vitrification (35), endometrium preparation (36), and endometrial thickness (37). After logistic regression analysis, we found that obstetrical history (parity), basal FSH level, and endometrial preparation regimens were independent risk factors for neonatal birth weight.

In our present study, we found that delayed blastocyst transfer did not pose adverse effects on neonatal birth weight. Therefore, it was reasonable to speculate that the growth potential of blastocysts could affect blastocysts at the early preimplantation period. The implantation process might eliminate blastocysts with lower adaptation capacity, thus theoretically selecting blastocysts with better growth potential. Several studies have illustrated that embryos with comparable developmental potential of cell division may eliminate a genetically abnormal cell line (38, 39).

In the present study, we focused on the effects of FET on singleton births based on a large sample, which ruled out the possibility of adverse fetal growth caused by some other factors, including twins and related pregnancy complications, and the possibility of adverse fetal growth caused by high estrogen levels in fresh cycles. Indeed, we acknowledged certain limitations in the present study. First, the present study was limited by the bias inherent in its retrospective nature. Despite adequate control for confounding factors, unavailable or unknown confounders may generate bias because of the retrospective design. Although anti-Mullerian hormone levels (AMH), thyroid-stimulating hormone (TSH), and polycystic ovary syndrome (PCOS) might be related to neonatal birth and endometrial thickness of HCG day (the day of HCG administration day) these variables were not available or had extensive missing data (>50%), and could not be included in this study. Second, in some cycles, top-quality embryos on D3 were transferred as a priority, while the morphologically poorer embryos were placed in extended culture until the blastocyst stage. The blastocyst quality was graded before being frozen, which might bias the difference. Third, most of the neonatal outcomes were accessed by telephone interview, which might result in underestimated birth defect rates. Finally, we showed that the rate of cesarean section was higher in the D6 group compared with the D5 group, while no statistically significant differences were found in the analysis of gestational age between the two groups. Moreover, there might be other factors that were not included in this study since the database was not fully completed. Further studies should investigate the mechanisms underlying the effect of delayed blastulation on neonatal outcomes.

## Conclusions

In summary, neonatal birth weight and the proportion of LGA, SGA, macrosomia, or LBW were similar between the D5 and D6 groups. We concluded that frozen and delayed blastocysts would not affect neonatal birth weight. However, further large prospective studies should be carried out to confirm these results and the underlying mechanisms should also be investigated.

## Data Availability Statement

The original contributions presented in the study are included in the article/supplementary material. Further inquiries can be directed to the corresponding author.

## Ethics Statement

The studies involving human participants were reviewed and approved by Hospital’s Ethics Committee for Reproductive Medicine. Written informed consent for participation was not required for this study in accordance with the national legislation and the institutional requirements.

## Author Contributions

JY contributed significantly to analysis and wrote the manuscript. ZW performed the data analyses and manuscript preparation. HC, LL, QY and HX helped perform the analysis with constructive discussions. RT contributed to the conception of the study. All authors contributed to the article and approved the submitted version.

## Funding

This study was supported by the Shandong Province Medical and Health Technology Development Project (2016WS0442) and the National Key Research and Development Program of China (2017YFC10010004).

## Conflict of Interest

The authors declare that the research was conducted in the absence of any commercial or financial relationships that could be construed as a potential conflict of interest.

## Publisher’s Note

All claims expressed in this article are solely those of the authors and do not necessarily represent those of their affiliated organizations, or those of the publisher, the editors and the reviewers. Any product that may be evaluated in this article, or claim that may be made by its manufacturer, is not guaranteed or endorsed by the publisher.
